# Supplementary material for: HIVprotI: an integrated web based platform for prediction and design of HIV proteins inhibitors
Source: J Cheminform. 2018 Mar 9;10:12. doi: 10.1186/s13321-018-0266-y (PMC5845081; doi:10.1186/s13321-018-0266-y)
Supplement: Supplementary file 2 — Additional file 2. Source code of HIVProtI web server. [file 13321_2018_266_MOESM2_ESM.zip › HIVprotI_Source-code/hivprotI/weka-3-6-6/doc/weka/core/CheckOptionHandler.html]

CheckOptionHandler


---


|  |  |  |
| --- | --- | --- |
| **Overview** \| **Package** \| **Class** \| **Tree** \| **Deprecated** \| **Index** \| **Help** \| **Weka's home** | |  |
| **PREV CLASS**   **NEXT CLASS** | **FRAMES**    **NO FRAMES**     **All Classes** |  |
| SUMMARY: NESTED \| FIELD \| CONSTR \| METHOD | DETAIL: FIELD \| CONSTR \| METHOD |  |


---


## weka.core Class CheckOptionHandler

```
java.lang.Object
  weka.core.Check
      weka.core.CheckOptionHandler
```

**All Implemented Interfaces:**: OptionHandler, RevisionHandler

---

``` public class CheckOptionHandler extends Check ```

Simple command line checking of classes that implement OptionHandler.

Usage:

 `CheckOptionHandler -W optionHandlerClassName -- test options` 


Valid options are:

```
 -D
  Turn on debugging output.
```

```
 -S
  Silent mode - prints nothing to stdout.
```

```
 -W
  Full name of the OptionHandler analysed.
  eg: weka.classifiers.rules.ZeroR
  (default weka.classifiers.rules.ZeroR)
```

```
 Options specific to option handler weka.classifiers.rules.ZeroR:
```

```
 -D
  If set, classifier is run in debug mode and
  may output additional info to the console
```

Options after -- are used as user options in testing the
OptionHandler

**Version:**
:   $Revision: 1.13 $

**Author:**
:   Len Trigg (trigg@cs.waikato.ac.nz), FracPete (fracpete at waikato dot ac dot nz)

---

**Constructor Summary**

`CheckOptionHandler()`


| **Method Summary** | |
| --- | --- |
| `boolean` | `checkCanonicalUserOptions()`             checks whether the user-supplied options stay the same after settting, getting and re-setting again |
| `boolean` | `checkDefaultOptions()`             checks whether the default options can be processed completely or some invalid options are returned by the getOptions() method. |
| `boolean` | `checkListOptions()`             checks whether the listOptions method works |
| `boolean` | `checkRemainingOptions()`             checks whether the user-supplied options can be processed completely or some "left-over" options remain |
| `boolean` | `checkResettingOptions()`             checks whether the optionhandler can be re-setted again to default options after the user-supplied options have been set. |
| `boolean` | `checkSetOptions()`             checks whether the user-supplied options can be processed at all |
| `void` | `doTests()`             Runs some diagnostic tests on an optionhandler object. |
| `OptionHandler` | `getOptionHandler()`             Get the OptionHandler used in the tests. |
| `java.lang.String[]` | `getOptions()`             Gets the current settings of the CheckClassifier. |
| `java.lang.String` | `getRevision()`             Returns the revision string. |
| `boolean` | `getSuccess()`             returns the success of the tests |
| `java.lang.String[]` | `getUserOptions()`             Gets the current user-supplied options (creates a copy) |
| `java.util.Enumeration` | `listOptions()`             Returns an enumeration describing the available options. |
| `static void` | `main(java.lang.String[] args)`             Main method for using the CheckOptionHandler. |
| `void` | `setOptionHandler(OptionHandler value)`             Set the OptionHandler to work on.. |
| `void` | `setOptions(java.lang.String[] options)`             Parses a given list of options. |
| `void` | `setUserOptions(java.lang.String[] value)`             Sets the user-supplied options (creates a copy) |

**Methods inherited from class weka.core.Check**

`getDebug, getSilent, setDebug, setSilent`

**Methods inherited from class java.lang.Object**

`equals, getClass, hashCode, notify, notifyAll, toString, wait, wait, wait`

**Constructor Detail**

### CheckOptionHandler

```
public CheckOptionHandler()
```


**Method Detail**

### listOptions

```
public java.util.Enumeration listOptions()
```

:   Returns an enumeration describing the available options.

    :   **Specified by:**: `listOptions` in interface `OptionHandler` **Overrides:**: `listOptions` in class `Check`
    :   **Returns:**: an enumeration of all the available options.

---


### setOptions

```
public void setOptions(java.lang.String[] options)
                throws java.lang.Exception
```

:   Parses a given list of options.


    Valid options are:

    ```
     -D
      Turn on debugging output.
    ```

    ```
     -S
      Silent mode - prints nothing to stdout.
    ```

    ```
     -W
      Full name of the OptionHandler analysed.
      eg: weka.classifiers.rules.ZeroR
      (default weka.classifiers.rules.ZeroR)
    ```

    ```
     Options specific to option handler weka.classifiers.rules.ZeroR:
    ```

    ```
     -D
      If set, classifier is run in debug mode and
      may output additional info to the console
    ```

    :   **Specified by:**: `setOptions` in interface `OptionHandler` **Overrides:**: `setOptions` in class `Check`
    :   **Parameters:**: `options` - the list of options as an array of strings **Throws:**: `java.lang.Exception` - if an option is not supported

---


### getOptions

```
public java.lang.String[] getOptions()
```

:   Gets the current settings of the CheckClassifier.

    :   **Specified by:**: `getOptions` in interface `OptionHandler` **Overrides:**: `getOptions` in class `Check`
    :   **Returns:**: an array of strings suitable for passing to setOptions

---


### setOptionHandler

```
public void setOptionHandler(OptionHandler value)
```

:   Set the OptionHandler to work on..

    :   **Parameters:**: `value` - the OptionHandler to use.

---


### getOptionHandler

```
public OptionHandler getOptionHandler()
```

:   Get the OptionHandler used in the tests.

    :   **Returns:**: the OptionHandler used in the tests.

---


### setUserOptions

```
public void setUserOptions(java.lang.String[] value)
```

:   Sets the user-supplied options (creates a copy)

    :   **Parameters:**: `value` - the user-supplied options to use

---


### getUserOptions

```
public java.lang.String[] getUserOptions()
```

:   Gets the current user-supplied options (creates a copy)

    :   **Returns:**: the user-supplied options

---


### getSuccess

```
public boolean getSuccess()
```

:   returns the success of the tests

    :   **Returns:**: true if the tests were successful

---


### checkListOptions

```
public boolean checkListOptions()
```

:   checks whether the listOptions method works

    :   **Returns:**: index 0 is true if the test was passed, index 1 is always false

---


### checkSetOptions

```
public boolean checkSetOptions()
```

:   checks whether the user-supplied options can be processed at all

    :   **Returns:**: index 0 is true if the test was passed, index 1 is always false

---


### checkDefaultOptions

```
public boolean checkDefaultOptions()
```

:   checks whether the default options can be processed completely
    or some invalid options are returned by the getOptions() method.

    :   **Returns:**: index 0 is true if the test was passed, index 1 is always false

---


### checkRemainingOptions

```
public boolean checkRemainingOptions()
```

:   checks whether the user-supplied options can be processed completely
    or some "left-over" options remain

    :   **Returns:**: index 0 is true if the test was passed, index 1 is always false

---


### checkCanonicalUserOptions

```
public boolean checkCanonicalUserOptions()
```

:   checks whether the user-supplied options stay the same after settting,
    getting and re-setting again

    :   **Returns:**: index 0 is true if the test was passed, index 1 is always false

---


### checkResettingOptions

```
public boolean checkResettingOptions()
```

:   checks whether the optionhandler can be re-setted again to default
    options after the user-supplied options have been set.

    :   **Returns:**: index 0 is true if the test was passed, index 1 is always false

---


### doTests

```
public void doTests()
```

:   Runs some diagnostic tests on an optionhandler object. Output is
    printed to System.out (if not silent).

    :   **Specified by:**: `doTests` in class `Check`

---


### getRevision

```
public java.lang.String getRevision()
```

:   Returns the revision string.

    :   **Returns:**: the revision

---


### main

```
public static void main(java.lang.String[] args)
```

:   Main method for using the CheckOptionHandler.

    :   **Parameters:**: `args` - the options to the CheckOptionHandler


---


|  |  |  |
| --- | --- | --- |
| **Overview** \| **Package** \| **Class** \| **Tree** \| **Deprecated** \| **Index** \| **Help** \| **Weka's home** | |  |
| **PREV CLASS**   **NEXT CLASS** | **FRAMES**    **NO FRAMES**     **All Classes** |  |
| SUMMARY: NESTED \| FIELD \| CONSTR \| METHOD | DETAIL: FIELD \| CONSTR \| METHOD |  |


---
